# Deletion of amelotin exons 3–6 is associated with amelogenesis imperfecta

**DOI:** 10.1093/hmg/ddw203

**Published:** 2016-07-12

**Authors:** Claire E.L. Smith, Gina Murillo, Steven J. Brookes, James A. Poulter, Sandra Silva, Jennifer Kirkham, Chris F. Inglehearn, Alan J. Mighell

**Affiliations:** 1 Leeds Institute of Biomedical and Clinical Sciences, St James’s University Hospital, University of Leeds, Leeds LS9 7TF, UK,; 2 Department of Oral Biology, St James's University Hospital, University of Leeds, Leeds LS9 7TF, UK,; 3 University of Costa Rica, School of Dentistry, San Pedro, Costa Rica,; 4 University of Costa Rica, Molecular Biology Cellular Centre (CBCM), San Pedro, Costa Rica and; 5 School of Dentistry, University of Leeds, Leeds LS2 9LU, UK

## Abstract

Amelogenesis imperfecta (AI) is a heterogeneous group of genetic conditions that result in defective dental enamel formation. Amelotin (AMTN) is a secreted protein thought to act as a promoter of matrix mineralization in the final stage of enamel development, and is strongly expressed, almost exclusively, in maturation stage ameloblasts. *Amtn* overexpression and *Amtn* knockout mouse models have defective enamel with no other associated phenotypes, highlighting *AMTN* as an excellent candidate gene for human AI. However, no *AMTN* mutations have yet been associated with human AI. Using whole exome sequencing, we identified an 8,678 bp heterozygous genomic deletion encompassing exons 3-6 of *AMTN* in a Costa Rican family segregating dominant hypomineralised AI. The deletion corresponds to an in-frame deletion of 92 amino acids, shortening the protein from 209 to 117 residues. Exfoliated primary teeth from an affected family member had enamel that was of a lower mineral density compared to control enamel and exhibited structural defects at least some of which appeared to be associated with organic material as evidenced using elemental analysis. This study demonstrates for the first time that *AMTN* mutations cause non-syndromic human AI and explores the human phenotype, comparing it with that of mice with disrupted *Amtn* function.

## Introduction

Amelogenesis is the formation of dental enamel, which consists predominantly of hydroxyapatite (HA) crystals (1). The process has three contiguous developmental stages that ultimately result in the most highly mineralised mammalian tissue, with distinctive structure, high mechanical strength and the ability to maintain function over a lifetime without cellular repair.

During the secretory stage, a proteinaceous extracellular matrix is incrementally laid-down by epithelially-derived ameloblasts via specialised cellular extensions termed Tomes’ processes. This matrix partially mineralises to delineate the enamel architecture which is characterised by bundles of enamel crystals (enamel prisms or rods) interdigited with interprismatic enamel crystals (2). As the enamel layer approaches full thickness, the Tomes’ process is retracted and the final outer enamel layer is aprismatic. The ameloblasts then enter the transition stage, characterised by reduced matrix protein secretion and internal reorganization (3). The cells then enter the maturation stage and begin secreting the serine protease KLK4 that completely degrades the proteinaceous matrix, leaving the immature enamel crystals bathed in enamel tissue fluid (4). Concomitantly, maturation stage cells cycle between so called rough and smooth ended ameloblasts; the former associated with acidification of the enamel microenvironment and massive active transport of mineral ions into the enamel and the latter associated with alkalination of the enamel microenvironment and protein readsorption (5). The significant increase in the active transport of mineral ions into the matrix during this stage accelerates enamel crystal growth in both width and thickness until the tissue volume is eventually occluded by mineral (6). Finally, the ameloblasts reduce, undergo apoptosis and on tooth eruption, are completely lost (7).

Amelogenesis imperfecta (AI) (MIM 104530) is a group of genetic conditions that result from defective amelogenesis. The enamel produced can be hypoplastic or hypomineralised due to inadequate enamel volume or mineralization, respectively. Mixed phenotypes also occur. The prevalence of AI is reported to be between 1/700 and 1/14,000 depending upon the population studied (8,9). The effect of AI on patients’ well-being is profound and the clinical care required is both challenging and costly (10).

AI may present in isolation or may be a component of syndromic disease. Autosomal recessive, dominant and X-linked forms of non-syndromic AI are recognised and mutations in many genes have already been shown to be causative (11–25). In addition to the known genes, there are a number of strong candidate genes implicated in AI based on their known function, pattern of expression and/or animal model studies. Foremost among these is the gene encoding amelotin (*AMTN*: MIM*610912) (26).

*Amtn* was first cloned in rodents during studies to identify proteins secreted by the enamel organ (26,27). It is found within a conserved enamel gene cluster located on chromosome 5 in mice, chromosome 14 in rats and chromosome 4 in humans. The cluster also includes the genes encoding enamelin (MIM*606585) and ameloblastin (MIM*601259), mutations in which are already known to cause AI (14,25). *Amen* encodes a 209 amino-acid protein rich in proline, leucine, threonine, glutamine, and glycine, with an N-terminal signal sequence that, once cleaved, gives rise to a mature 20.4 kDa secreted protein (27,28). AMTN has been shown to be restricted to the basal lamina of maturation stage ameloblasts, the structure that links ameloblasts with the developing enamel (29). A functional study by Abbarin and colleagues suggested that AMTN promotes HA precipitation, serving a critical role in the formation of the final compact aprismatic enamel surface layer during the maturation stage of amelogenesis (30).

Consistent with these findings, both an *Amtn* overexpression mouse model in which AMTN was over-expressed under the control of an *Amel* promoter (p*Amel*:*Amtn*^+/+^), and a knockout (*Amtn*^-/-^) mouse model, have defective enamel (31,32). The p*Amel*:*Amtn*^+/+ ^mouse model has brittle enamel that is thinner than wild-type (WT), with a defective surface layer (31). By contrast the *Amtn^-/-^* mouse model has mandibular incisors with a chalky appearance and rough, irregular surface enamel that is easily chipped away, though maxillary incisors and molars were unaffected, as were *Amtn*^+/-^ mice (32).

These observations have made *AMTN* a strong candidate gene for involvement in human AI, as noted by a number of research groups (30,33,34). To date, however, no human mutations have been reported. Here we report the first human *AMTN* mutation and show that the corresponding human phenotype is non-syndromic dominant hypomineralised AI.

## Results

### Patient phenotype

**Figure 1.**
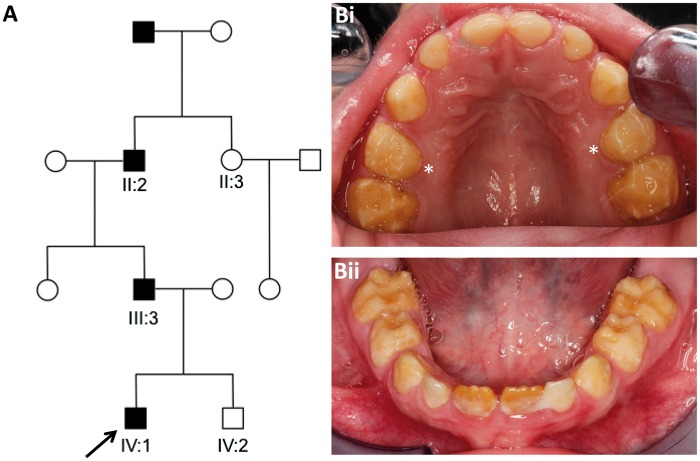
Family pedigree and dental phenotype. (**A**) Pedigree of the Costa Rican family investigated. Affected family members are shaded. (**B**) The mixed dentition of the index case, IV:1, aged 5 years (arrow on pedigree) was characterised by generalised hypomineralised AI with some hypoplasia involving all teeth. There was post-eruptive enamel loss with retention of a thin band of enamel at the cervical margin (examples marked with ∗). Periodontal health was consistent with that expected for patient age in each of the affected individuals with nothing to suggest a co-segregating phenotype.

We identified a Costa Rican family segregating autosomal dominant hypomineralised AI in the absence of any co-segregating health problems (Fig. 1 and Supplementary Material, Fig. S2).

### Whole-exome sequencing

In order to identify the cause of AI in this family, we performed whole exome sequencing (WES) on DNA from individuals II:2, II:3, III:3 and IV:1. Following alignment, processing and duplicate removal, a mean depth of 60.06, 89.77, 66.44 and 110.54 reads per base were observed, respectively for individuals II:2, II:3, III:3 and IV:1 with 98.9%, 99.6%, 98.7% and 99.8% of the bases covered by at least 5 reads respectively (further alignment statistics are available in Supplementary Material, Table S1). Indel and single nucleotide variants were called in variant call format (VCF) using the Haplotype Caller function of the Genome Analysis Toolkit (GATK) (35).

In light of the autosomal dominant inheritance in the family (confirmed by male-to-male transmission) and relative rarity of AI, variants present in the National Centre for Biotechnology Information’s dbSNP142 (dbSNP142; http://www.ncbi.nlm.nih.gov/projects/SNP/), the National Heart, Lung and Blood Institute’s Exome Sequencing Project’s Exome Variant Server (EVS; http://evs.gs.washington.edu/EVS/) or the Exome Aggregation Consortium v0.3 (ExAC; http://exac.broadinstitute.org/) with minor allele frequencies equal to or greater than 0.1% were excluded, as were homozygous variants and those located on the X chromosome. The resulting variants were then filtered to select those present in all three affected individuals (II:2, III:3 and IV:1) but not in the unaffected individual (II:3). This list was further filtered to exclude all changes other than missense, frameshift or stop mutations, exonic insertions/deletions or variants located at splice consensus sites. This left a total of 26 variants with a Combined Annotation Dependent Depletion (CADD) pathogenicity score (v1.3) of over 15 (Supplementary Material, Table S2). Alongside variant calling, Copy Number Variant (CNV) analysis was also performed, using ExomeDepth software (36). This compares read depths across all captured exons of samples from affected individuals (II:2, III:3 and IV:1) and the unaffected individual (II:3) against the read depths of between 5 to 10 samples from unrelated individuals whose DNA had been processed within the same WES batches, using identical conditions, as the affected samples from the family.

None of the 26 potentially pathogenic variants identified were in genes known to be involved in AI or implicated in AI by function, expression or animal model studies. However, a heterozygous deletion spanning exons 3 to 6 of *AMTN* (chr4:71388473-71394475, GRCh37) was detected by the CNV analysis software (Supplementary Material, Table S3 and Fig. S1). While large CNVs are not reported in variant databases such as dbSNP and ExAC, this deletion was not present in the Database of Genomic Variants (37).

### PCR and Sanger sequencing

**Figure 2.**
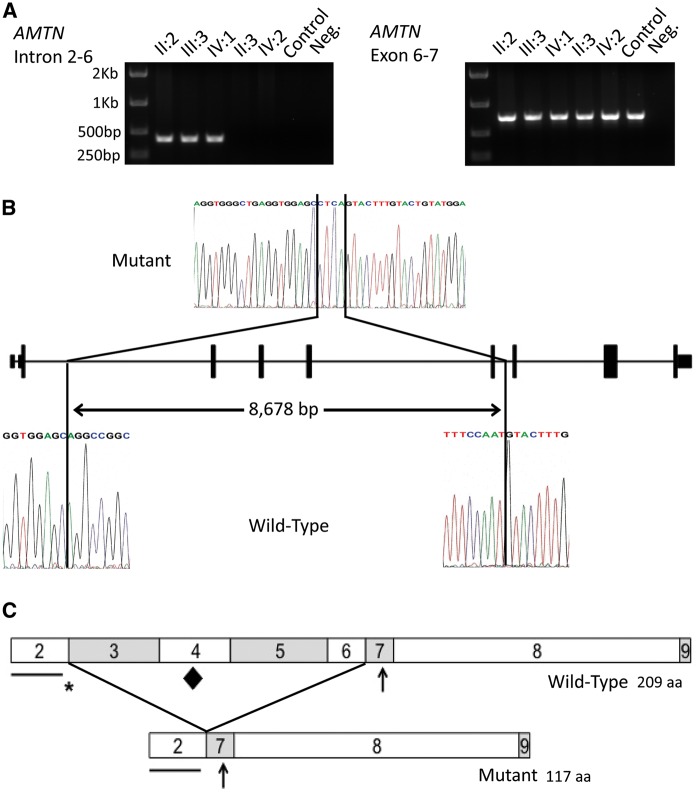
Genotyping of the mutation, identification of the deletion breakpoints and a schematic diagram of the predicted effect of the deletion on the AMTN protein. (**A**) PCR analysis of the heterozygous deletion of exons 3 to 6 of *AMTN* in the members of the family investigated. Amplification using primers spanning introns 3 to 6, designed to produce a product of 9,073 bp for the wild-type allele, produced a product of 399 bp in affected individuals confirming a deletion encompassing exons 3 to 6. Amplification of exons 6 to 7 was observed in all individuals. (**B**) Sanger sequencing electropherograms of mutant and wild-type alleles to identify the deletion breakpoints. Comparison of the mutant sequence with a control sequence revealed a deletion of 8,678 bp spanning exons 3 to 6 of *AMTN* and an insertion of 4 bp. (**C**) Schematic diagrams of the wild-type and the predicted mutant *AMTN* protein structures. The predicted mutant AMTN protein lacks the amino acid sequence encoded by exons 3 to 6, resulting in the loss of 92 amino acids. The contribution of each exon to the protein is signified by the boxes labelled with the number corresponding to the exon number. Important motifs are labelled with symbols as follows: the line shows the position of the putative signal peptide, encoded by exon 2, the asterisk shows the position of the LPQ motif, encoded by exons 2 and 3, the diamond shows the position of the IPLT motif, encoded by exon 4, predicted to be an O-glycosylation site. The arrow shows the position of the SXE phosphorylation motif, encoded by exon 7.

In order to investigate the potential heterozygous deletion further, a long range PCR assay was undertaken using primers designed to amplify introns 2 to 6 (Supplementary Material, Table S4). For WT DNA, the product size was predicted to be 9,073 bp, but in affected individuals only a product of approximately 400 bp was observed. The large predicted WT band was never observed in controls nor in patients, probably due to its large size. In contrast, PCR amplification using primers flanking exons 6 and 7 produced a band of the expected size (713 bp) in all individuals (Fig. 2A).

Sequencing of the introns 2 to 6 product from affected individuals confirmed the presence of a heterozygous deletion within *AMTN* spanning a total of 8,678 bp, accompanied by a 4 bp insertion (chr4:71,385,896-71,394,573delinsCTCA; GRCh37). The deletion encompasses all of the 276 bp of exons 3-6 plus 2,576 bp of 5′ genomic sequence and 98 bp of 3′ sequence (c.54 + 1347_330 + 98delinsCTCA (NM_212557, Fig. 2B)). The heterozygous deletion was present in all available affected family members (II:2, III:3 and IV:1) and absent from all unaffected family members tested (II:3 and IV:2). This deletion is predicted to create an in-frame deletion of 92 amino acids (p.Q19_Q110del, NP_997722), shortening the protein from 209 to 117 amino acids (Fig. 2C).

In an attempt to determine the consequences of loss of exons 3-6 on transcription of *AMTN* mRNA, leukocyte cDNA was obtained from an affected individual (III:3). While cDNA amplification with control primers (*P53*) was successful (data not shown), amplification of *AMTN* products failed in both patient and control cDNA. This is consistent with the reported tissue specific expression pattern of *Amtn*, (26). As a consequence, there is no detectable expression of *AMTN* mRNA in blood, a known issue for enamel specific transcripts (25).

Based on this result, we screened all coding exons and flanking introns of *AMTN* in an additional 35 dominant AI samples, but no single nucleotide variants or small indels were identified (Supplementary Material, Table S5). We also screened the cohort for the deletion identified in the family presented here, using the primer pair designed to amplify introns 2 to 6, but did not identify any further families that carried the same deletion.

### Enamel phenotyping

On clinical examination of the teeth, the three family members heterozygous for the mutation (II:2, III:3 and IV:1) exhibited hypomineralised AI (Fig. 1). Individuals II:3 and IV:2 did not carry the mutation and exhibited a normal dentition (Supplementary Material, Fig. S2).

**Figure 3.**
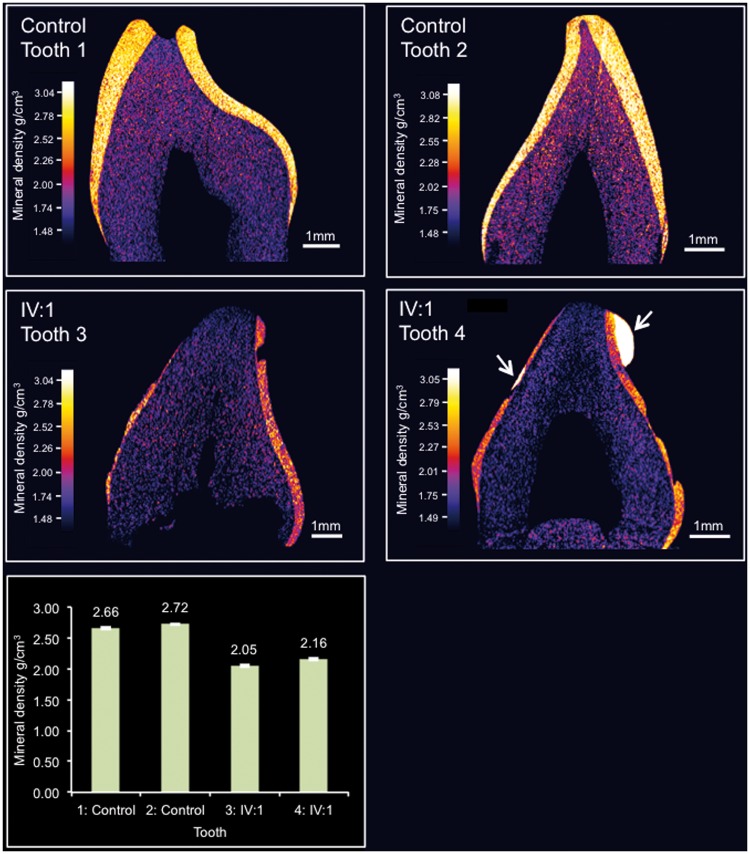
High resolution X-ray CT analysis of exfoliated teeth from control individual and individual IV:1. Typical CT sections through the teeth are presented using false colour calibrated with respect to mineral density to generate mineral density maps. Mean enamel mineral density for each tooth is also shown graphically, error bars represent the twice the standard error of the mean. The teeth from the control individuals (Teeth 1 and 2) exhibit an enamel layer apparently normal in structure and density. Teeth 3 and 4 from IV:1 exhibit an obvious enamel covering though it is thinner, chipped, absent in places and is reduced in mineral density compared with teeth 1 and 2. Note that tooth 4 has undergone restoration (indicated by arrows). Videos of 3D rendered CT data showing surface detail and internal structure of teeth 1-4 are available as Supplementary Material.

Two deciduous teeth from IV:1 were available for phenotypic characterization after natural exfoliation. High resolution-computerised tomography (CT) scanning (Fig. 3 and supplemental videos 1-4) of control teeth 1 and 2 and teeth 3 and 4 from IV:I, showed that teeth 3 and 4 exhibited reduced enamel mineral density. The region of tooth 4 that had undergone restoration was excluded from the analysis. Enamel thickness also appeared to be reduced though this was difficult to confirm due to the possibility of post-eruptive tissue loss. The enamel mineral densities of teeth 3 and 4 were 2.05 g/cm^3^ +/-0.02 g/cm^3^ (2x standard error of the mean) and 2.16 g/cm^3^ +/-0.02 g/cm^3^ respectively, compared to 2.66 g/cm^3^ +/-0.02 g/cm^3^ and 2.72 +/-0.06 g/cm^3^ for the two control teeth, 1 and 2. For reference, a range of 2.69-2.92 g/cm^3^ has been previously reported for deciduous enamel (38).

**Figure 4.**
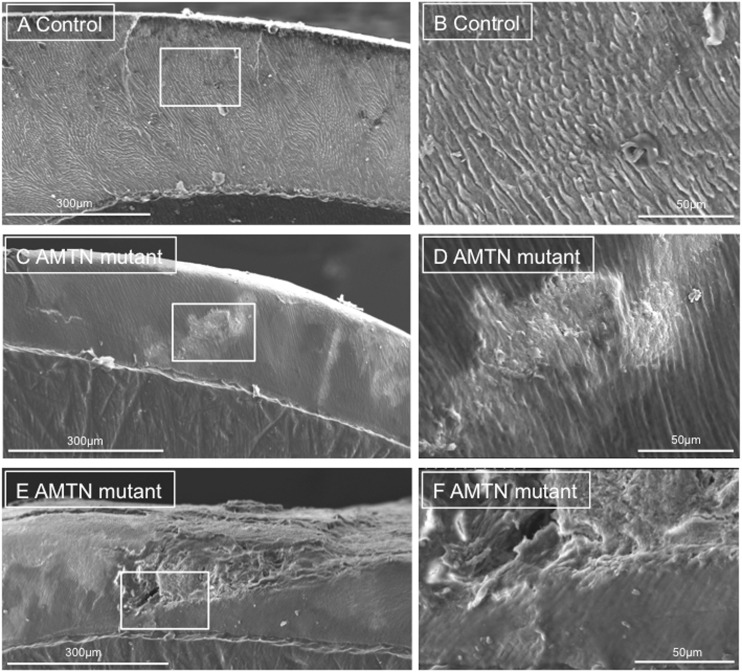
SEM of representative exfoliated teeth. (**A and B**) SEM of tooth 1 from the control individual, (**C–F**) SEM of tooth 4 from individual IV:1, boxed regions on pictures A, C and E reflect the boundaries of the photographs taken at higher power in these regions, labelled B, D and F. Control tooth 1 exhibits normal, typical enamel architecture comprising prisms (rods) of individual enamel crystallites. Tooth 4 exhibits both regions of relatively normal enamel and disturbed structure. The cross sectional surface of Tooth 4 has a “smooth” appearance that may reflect the presence of organic material.

On scanning electron microscopy (SEM), control tooth 1 (Fig. 4A and B) exhibited prismatic structure characteristic of normal human enamel. Tooth 4 from individual IV:1 also exhibited areas of prismatic enamel though the fine detail of the ultrastructure was obscured by what appeared to be overlying material (Fig. 4C and D). The enamel also displayed distinct regions where the enamel structure was particularly disturbed; the prismatic structure having a “gnarled” appearance (Fig. 4E and F). These regions spanned the entire enamel and were not confined to the outermost enamel layers. In some areas of the tooth, the enamel was missing, perhaps indicative of post eruptive failure under masticatory stress.

In order to determine whether the material that appeared to be overlying the enamel of tooth 4 from individual IV:1 was of organic origin, we treated the tooth with 6% sodium hypochlorite which is well known for its ability to solubilise (oxidise) a range of organic materials such as proteins, lipids and nucleic acids. Overlying material was still obvious in the SEM and therefore apparently resistant to hypochlorite treatment (Supplementary Material, Fig. S3).

Elemental analysis by energy dispersive X-ray spectroscopy (EDX; Supplementary Material, Table S6 and Fig. S4), carried out prior to the hypochlorite treatment, showed that control tooth 1 consisted primarily of elements expected to be found in enamel, which is predominantly comprised of a substituted calcium hydroxyapatite (1,39). Atomic mass percentage contributions showed that control tooth A enamel consisted of 57.2% oxygen, 19.9% calcium, 12.3% carbon and 10.1% phosphorus, with the remaining 0.45% being sodium. In contrast, tooth 4 enamel (individual IV:1) showed varying elemental content depending upon the area of enamel analysed. All areas of tooth 4 had higher carbon and lower oxygen percentage content than control tooth 1. Three of the four enamel areas analysed for tooth 4 also had a lower phosphorus percentage content than control tooth 1. Furthermore, three areas of tooth 4 enamel contained nitrogen (Area 1: 11.1%, area 2: 11.6% and area 4: 7.2%) which was not present in enamel from control tooth 1.

EDX elemental analysis of tooth 4 enamel (individual IV:1) following hypochlorite treatment (Supplementary Material, Table S7 and Fig. S5) showed that the atomic mass percentages of nitrogen and carbon were apparently reduced. This suggests that some organic material may have been removed by the hypochlorite treatment but the continued presence of an overlying layer suggests that the material is comprised, in part at least, of material that is resistant to hypochlorite.

## Discussion

We identified a family from Costa Rica segregating autosomal dominant hypomineralised AI. WES analysis of rare variants and CNVs in known and candidate AI genes revealed a reduction in the number of reads across *AMTN* exons 3 to 6. Further investigation confirmed this to be an 8,678 bp deletion encompassing exons 3-6 and flanking intronic sequence. PCR analysis showed that the deletion segregates with the disease phenotype in all available family members. A screen of 35 additional dominant AI families revealed no further mutations.

*AMTN* consists of nine exons, of which eight (exons 2 to 9) are protein coding. No transcript could be amplified from patient blood, so it was not possible to determine the precise effect of the loss of exons 3-6 on the mRNA produced. One hypothesis is that the deletion leads to the production of a mature transcript containing exons 1, 2, 7, 8 and 9. This is a strong possibility as the relevant splice consensus sequences remain intact and the exons flanking those deleted are in phase zero, meaning that the deletion would leave any translated protein in frame (40). For this reason the transcript is unlikely to be subject to nonsense-mediated decay (41).

Previous studies have found that *AMTN* exon 2 encodes the putative signal peptide and exon 7 encodes a consensus SXE phosphorylation motif targeted by the casein kinase FAM20C (Family With Sequence Similarity 20, Member C, MIM*611061) (27,34). Assuming that a mutant transcript and protein are produced, the deletion identified in the family described here would not remove either of these highly conserved regions (34). However, deletion of exons 3-6 would disrupt a conserved, amino terminal, IPV like, tripeptide motif, LPQ, encoded by the last six bases of exon 2 and the first three bases of exon 3 respectively. This motif is thought to be responsible for the efficient trafficking of acidic proteins (the calculated pI of unmodified human AMTN is 5.29) out of the endoplasmic reticulum (ER) (42). Mutations affecting the IPV motif of the dentin matrix protein, dentin sialophosphoprotein (MIM *125485), are known to result in dominant negative dentinogenesis imperfecta type I (MIM #125490) due to a failure to traffic the protein out of the ER and the associated retention of WT protein (43). The deletion identified in this family, if expressed, will replace the polar glutamine residue encoded by the first three bases of exon 3 with non polar glycine encoded by the first three bases of exon 7. The deletion would also delete an IPLT motif in exon 4 (34), which is predicted to be an O-glycosylation site and is therefore likely to be important for AMTN function (44). Moreover, the deletion of a significant proportion of a protein or defective covalent modification may cause misfolding leading to ER stress driven cell pathologies. Rat *Amtn* transcript variants lacking exons 3 to 7 have been identified (27) suggesting that amelotin lacking the IPV-like tripeptide motif and the O-glycosylation site may still have some functional role and that the protein is tolerated during secretory trafficking. However, the expression level of this truncated *Amtn* transcript is unknown and it may be that it is expressed at very low levels only.

Pseudogenization of *AMTN* in enamel-less species has meant that AMTN is regarded as an enamel-specific protein (34). It has been proposed that prismatic enamel evolved in mammals concurrently with AMTN expression becoming restricted to the maturation stage and with changes to *AMTN* exon boundaries and splicing (34,45,46). The exact function of AMTN in mammals remains unknown, but its expression within the basal lamina of maturation stage ameloblasts and at the internal basal lamina within the junctional epithelium of erupted rodent teeth suggests functions in both enamel mineralization and in dento-gingival attachment (26,27). However, dento-gingival attachment appeared unaffected in both amelotin null (*Amtn^-/-^*) mice (32) and AI affected individuals carrying the heterozygous *AMTN* exon 3-6 deletion reported here.

In *Amtn^-/-^* mice (32,47), the effect on enamel phenotype varied depending on tooth type, with the continually erupting mandibular incisors most affected while molars appeared unaffected and maxillary incisors were merely stained. These differences may be related to the rate of formation/eruption of the different teeth; mandibular incisor enamel has less time in which to fully mineralise prior to eruption and AMTN may be required to ensure that the finished enamel surface is fully mineralised prior to enamel eruption. Enamel thickness (at eruption) and the characteristic decussating prismatic architecture were also apparently unaffected in *Amtn^-/-^* teeth, perhaps reflecting the fact that AMTN is not expressed during the secretory stage in WT animals (enamel thickness and rod architecture being determined during the secretory stage (48)). In the ensuing transition/maturation stages, where AMTN is maximally expressed and the enamel accrues the majority of its final mineral content, secondary mineralization was delayed; especially in the deeper enamel layers (32,47). Our findings on teeth from a patient heterozygous for the *AMTN* deletion showed a 18.8–24.6% reduction in enamel mineral density compared to control teeth, in contrast, the final mean enamel mineral density achieved across the full enamel thickness in *Amtn^-/-^* teeth was reduced by 2.1% and was not significantly different to WT (32,47). Microhardness values for the inner and middle enamel layers in null mice were also no different to WT but the microhardness of the surface layer in null mice was significantly reduced by approximately 23% (47). It was concluded that AMTN plays a crucial role in the production of a normally mineralised enamel surface in rodent mandibular incisors. Unfortunately, given that the human teeth characterised in this study were erupted and subject to wear in the mouth, it is not possible to comment specifically on the integrity or otherwise of the outer aprismatic enamel in the presence of this human *AMTN* mutation.

Elemental analysis of one of the teeth from a human patient carrying the *AMTN* mutation revealed consistently higher carbon and lower oxygen percentage content than control teeth. Also, nitrogen was present in three out of four regions analysed for the teeth from the human patient carrying the AMTN mutation but was undetected in the control tooth. These data suggested that organic material was present within the enamel and are consistent with the SEM appearance of the affected teeth which showed the presence of a layer of material obscuring the underlying fine structure of the enamel architecture. Hypochlorite treatment was used in an attempt to determine the nature of this overlying material but SEM revealed no obvious change in the appearance of the enamel, or the material, following treatment. However, analysis by EDX showed a reduction in the nitrogen and carbon content after treating the enamel with hypochlorite, suggesting that at least some of the organic material had been removed. We therefore conclude that the remainder was refractory to hypochlorite treatment, perhaps due to crosslinking or stabilization by interaction (or inclusion) with an inorganic calcium phosphate phase.

Unusually high levels of organic material of developmental origin have been previously reported to be present in hypomaturation forms of AI (49). Organic material of external origin (e.g. salivary protein and protein from dietary sources) have been detected in carious enamel (50) suggesting that disruption of the enamel surface may allow organic material to enter the enamel layer. In the affected tooth here, the organic material was not confined to the surface layers but was present throughout the enamel. However, we are unable to conclude whether the organic material detected entered the tissue post-eruption due to disturbances in the enamel structure or whether it is of developmental origin, representing retained matrix protein.

The fact that the reduction in mean enamel mineral density in the teeth of the human AI patient heterozygous for an *AMTN* deletion was much greater than that in *Amtn*^-/-^ mice, represents a clear phenotypic difference between the mouse model and the human phenotype characterised in this study. This may reflect the type of mutation present, since the human patients have an in frame deletion, predicted to produce a mutant transcript and possibly also a mutant protein whereas the mice have been shown not to produce any protein and to exhibit a 300 fold reduction in transcript levels (32). Núñez et al. reported that heterozygous *Amtn^+/-^* mice had no enamel defects and suggested that murine amelogenesis is unaffected even in the presence of half of the normal amount of amelotin (47). This suggests that the *AMTN* deletion identified here may have a dominant-negative (toxic gain of function) effect due to the presence of a mutated protein, rather than acting through haploinsufficiency.

Whether any toxic gain of function, associated with the 8,678 bp heterozygous genomic deletion reported here is manifest in the extracellular or intracellular compartments (or both) is unclear. Amelotin has been shown to interact with itself and with ODAM, leading to the hypothesis that it forms large aggregates that are integral to the structure and function of the basal lamina-like layer between the maturation stage ameloblasts and the enamel surface (51–53). In the present study, the whole enamel thickness is hypomineralized, suggesting that the mutant AMTN may: (i) Disrupt the normal function of the basal lamina-like layer compromising mineral ion ingress into the deeper enamel; and/or (ii) Impede the egress of degraded enamel matrix components out of the enamel impacting on the mineralization of the whole enamel layer (the “smooth” appearance of affected enamel seen in Figure 4C and D suggests the presence of organic material overlying the crystals and the nitrogen identified in EDX elemental analysis (Supplementary Material, Table S6 and Fig. S4) that was partially extractable using hypochlorite (Supplementary Material, Table S7 and Fig. S5) also supports this) and/or (iii) mis-fold, impairing trafficking through the ER, impacting directly on ameloblast function or causing cell death through ER stress in a manner similar to that described for a Y64H amelogenin mutation (54).

Given the long-standing status of *AMTN* as an AI candidate gene, it may seem surprising that mutations have not been reported previously. It is possible that in WES based studies, the type of mutation identified here may have been overlooked due to the bias inherent in this approach, which detects single nucleotide variants and smaller indels much more effectively than large indels or rearrangements. CNV analysis should therefore be performed on WES data as a standard approach alongside single/multiple nucleotide variant calling. Furthermore, the large exonic deletion reported here is also unusual in that it leaves the protein in the frame. It is possible that the majority of heterozygous *AMTN* mutations in human patients do not result in a toxic gain of function, but rather are functional *AMTN* knockouts, causing either no effect or a clinical phenotype so mild that they remain undiagnosed.

In summary, identification of a 8,678 bp heterozygous genomic deletion encompassing exons 3-6 of *AMTN* in a Costa Rican family confirms for the first time that *AMTN* mutations can cause autosomal dominant hypomineralized human AI in the absence of other obvious co-segregating clinical features. The mechanisms causing AI in this case are unclear, but the comparison between the enamel phenotype of exfoliated deciduous teeth from human patients with that of *Amtn*^-/-^ and *Amtn*^+/-^ mouse models suggests that the mouse models do not accurately reflect or predict the disease mechanism operating in this human case. Rather, toxic gain of function, associated with the mutant protein may drive pathology in these patients.

## Materials and Methods

### Patients

Affected individuals and family members were recruited following informed consent in accordance with the principles outlined by the declaration of Helsinki, with local ethical approval. Genomic DNA samples were obtained using Oragene® DNA sample collection kits (DNA Genotek, ONT, Canada) according to the manufacturer’s instructions.

### Whole exome sequencing and analysis

Three micrograms of genomic DNA were processed according to the Agilent SureSelect XT Library Prep protocol (Agilent Technologies, CA, US). Sure Select Human All Exon V5 (Agilent Technologies) was used as the capture reagent. Sequencing was performed using a 100 bp paired-end protocol on an Illumina HiSeq 2500 sequencer (Illumina, CA, USA). The resulting fastq files were aligned to the human reference genome (GRCh37) using Novalign software and processed in the SAM/BAM format using Picard (http://picard.sourceforge.net) and the GATK java programs (35). Exome depth was used for CNV analysis according to the developers’ guidelines (36).

All genomic coordinates are based on the GRCh37 human reference genome. The reference gene sequence upon which *AMTN* mutation nomenclature is based is RefSeq transcript NM_212557.

The variant identified in this study has been submitted to the Leiden Open Variant Database at http://dna2.leeds.ac.uk/LOVD/variant ID: 0000000172.

### Tooth phenotyping

Two deciduous teeth were available after natural exfoliation for phenotypic characterization. These were compared to two control teeth obtained from the Skeletal Tissues Research Bank (School of Dentistry, University of Leeds) with ethical approval and patient consent. The teeth were subjected to high resolution CT using a Skyscan 1172 (Brucker, UK) operated at 100 kV; source current of 100 µA, using an Al/Cu filter to reduce beam hardening. CT slices were reconstructed using Skyscan Recon software and rendered as 3D videos using Skyscan CTVox software. The CT images were calibrated using a calibration phantom comprising of three different HA standard of known mineral densities. Measurement of mean mineral density of the enamel of each tooth was carried out by first identifying regions of enamel using ImageJ software (http://imagej.nih.gov/ij/) and the Trainable Weka Segmentation plugin (http://fiji.sc/Trainable_Weka_Segmentation). The mean mineral density of the enamel was determined by measuring the mineral density of areas identified as enamel on every 10^th^ CT slice taken through each tooth. Calibrated colour contour maps of mineral density were also generated using ImageJ.

For SEM, teeth were sectioned with a diamond disk and the cut surfaces smoothed using fine carborundum paper. Specimens were etched in 30% phosphoric acid for 20 seconds followed by thorough rinsing in excess distilled water. Teeth were dried overnight under vacuum and sputter coated with gold. Specimens were observed using a Hitachi S-3400N scanning electron microscope (Hitachi, Tokyo, Japan) operated at an accelerating voltage of 15 kV.

For EDX, elemental analysis was performed on selected regions of the enamel using a detector fitted with an ultrathin window using Bruker Quantax Espirit software version 1.9.4 (Bruker). A minimum of nine measurements (maximum of thirty) were obtained for each specified enamel region. The mean composition of each enamel region was then calculated using atomic mass percentage measurements.

To investigate the effect of hypochlorite on the enamel, a fresh enamel surface was exposed by removing the gold coating with fine carborundum paper and acid etching as described above. After thorough rinsing in distilled water, the specimen was immersed in 6% sodium hypochlorite (Sigma, UK) for 10 minutes followed by thorough rinsing in distilled water. Teeth were dried, sputter coated and imaged and subjected to EDX elemental analysis as described above.

## Supplementary Material

Supplementary Material is available at *HMG* online.

Supplementary Data
