# Supplementary material for: Deletion of amelotin exons 3–6 is associated with amelogenesis imperfecta
Source: Hum Mol Genet. 2016 Jul 12;25(16):3578–87. doi: 10.1093/hmg/ddw203 (PMC5179951; doi:10.1093/hmg/ddw203)
Supplement: Supplementary Data [file supp_25_16_3578__index.html]

Deletion of amelotin exons 3–6 is associated with amelogenesis imperfecta — Deletion of amelotin exons 3–6 is associated with amelogenesis imperfecta — Supplementary Data 

# Deletion of amelotin exons 3–6 is associated with amelogenesis imperfecta

## Supplementary Data

files

- Supplementary Data - docx file
