# Supplementary material for: Deletion of amelotin exons 3–6 is associated with amelogenesis imperfecta
Source: Hum Mol Genet. 2016 Jul 12;25(16):3578–87. doi: 10.1093/hmg/ddw203 (PMC5179951; doi:10.1093/hmg/ddw203)
Supplement: Supplementary Data [file supp_ddw203_AMTNsupplementary5.docx]

**Supplementary data**

| **Sample** | **Capture reagent** | **Depth of coverage** | **% bases covered by >4 reads** | **% bases covered by >9 reads** | **% bases covered by >14 reads** | **% bases covered by >19 reads** | **% bases covered by >24 reads** | **% bases covered by >29 reads** |
| --- | --- | --- | --- | --- | --- | --- | --- | --- |
| II:2 | SSV5 | 60.06 | 98.9 | 97.6 | 95.4 | 91.9 | 87.1 | 81.3 |
| II:3 | SSV5 | 89.77 | 99.6 | 99.0 | 98.1 | 96.6 | 94.5 | 91.8 |
| III:3 | SSV5 | 66.44 | 98.7 | 97.3 | 94.9 | 91.6 | 87.3 | 82.2 |
| IV:1 | SSV5 | 110.54 | 99.8 | 99.4 | 98.8 | 97.8 | 96.5 | 94.8 |

**Table S1** Alignment statistics for whole exome sequencing.

Alignment statistics were generated using the regions targetted by the capture reagent: SSV5 - SureSelectXT Human All Exon V5.

| **Genomic variant (GRCh37)** | **dbSNP reference^a^** | **Amino acid change** | **Gene** | **CADD v1.3^b^** | **SIFT^c^** | **Polyphen^d^** | **RefSeq**  **transcript** |
| --- | --- | --- | --- | --- | --- | --- | --- |
| 1: 45471767 G>C | N/A | p.R583G | *HECTD3* | 32 | Deleterious  0 | Probably damaging 0.982 | NM_024602 |
| 1: 155028673 C>T | N/A | p..R288C | *ADAM15* | 32 | Deleterious  0 | Probably damaging 1 | NM_207197 |
| 20: 2796081 T>G | rs117426258 | p.L84R | *C20orf141* | 28.5 | Deleterious  0 | Possibly damaging 0.883 | NM_080739 |
| 10: 129681992 G>A | rs141771225 | p.P126L | *CLRN3* | 27.1 | Deleterious  0 | Probably damaging 1 | NM_152311 |
| 3: 49845339  T>C | rs561051731 | p.I849V | *UBA7* | 26.4 | Deleterious  0 | Probably damaging 0.999 | NM_003335 |
| 19: 12429806 C>A | N/A | p.G345C | *ZNF563* | 26.0 | Deleterious  0 | Probably damaging 0.999 | NM_145276 |
| 7: 55266431 T>C | N/A | p.M908T | *EGFR* | 25.5 | Deleterious  0.02 | Probably damaging 1 | NM_005228 |
| 1: 159899551 C>T | N/A | p.V735M | *IGSF9* | 25.1 | Deleterious  0 | Possibly damaging 0.632 | NM_001135050 |
| 18: 59221687 T>C | rs147507320 | p.V722A | *CDH20* | 24.9 | Deleterious  0.03 | Probably damaging 0.996 | NM_031891 |
| 7: 73038619 C>G | N/A | p.Q68H | *MLXIPL* | 23.9 | Tolerated 0.37 | Possibly damaging 0.62 | NM_032951 |
| 1: 167343355 C>T | rs375705900 | p.T138M | *POU2F1* | 23.8 | Tolerated 0.14 | Unknown 0 | NM_002697 |
| 1: 35650194 G>A | N/A | p.R663C | *SFPQ* | 23.4 | Tolerated 0.15 | Benign 0.217 | NM_005066 |
| 18: 33785103  CCT>C | N/A | p.? | *MOCOS* | 23.2 | N/A | N/A | NM_017947 |
| 20: 3145149 T>G | N/A | p.K612T | *LZTS3* | 23.3 | Tolerated 0.14 | Possibly damaging 0.859 | NM_001282533 |
| 20: 3145150 T>C | N/A | p.K612E | *LZTS3* | 23.1 | Tolerated 0.18 | Possibly damaging 0.801 | NM_001282533 |
| 1: 190234086 G>A | rs200575097 | p.T176M | *BRINP3* | 22.9 | Deleterious 0.03 | Benign 0.235 | NM_199051 |
| 6: 37605158 C>T | rs201115181 | p.A952T | *MDGA1* | 22.9 | Tolerated low confidence 0.19 | Benign 0.04 | NM_153487 |
| 2: 171641303 C>T | rs551957588 | p.P59L | *ERICH2* | 22.8 | Deleterious low confidence 0.01 | Benign 0.269 | NM_001289947 |
| 3: 75788040 G>A | rs111880168 | p.A195V | *ZNF717* | 22.6 | Tolerated 0.06 | Possibly damaging 0.739 | NM_0012890209 |
| 17: 48195540 ATCCGAGCCTTCACCATCC>A | N/A | p.E59_S64del | *SAMD14* | 22.1 | N/A | N/A | NM_174920 |
| 17: 48687281 G>A | N/A | p.A1582T | *CACNA1G* | 22.0 | Tolerated 0.41 | Benign 0.027 | NM_018896 |
| 11: 85435159 C>T | N/A | p.E1305K | *SYTL2* | 21.6 | N/A | Benign 0.005 | NM_001162953 |
| 20: 23472425 T>C | N/A | p.S41P | *CST8* | 21.5 | Deleterious 0 | Probably damaging 0.991 | NM_005492 |
| 1: 115226899 C>A | rs139582106 | p.Q189H | *AMPD1* | 18.62 | Tolerated 0.29 | Probably damaging 0.928 | NM_000036 |
| 6: 80341219 T>A | N/A | p.V14E | *SH3BGRL2* | 17.56 | Tolerated 0.38 | Benign 0.112 | NM_031469 |
| 11: 70172724 A>C | rs534856862 | p.S224R | *PPFIA1* | 15.91 | Tolerated 0.34 | Benign 0.035 | NM_003626 |

**Table S2** Details of the 26 genomic variants identified by WES after filtering.

Variants shared by II:2, III:3 and IV:1, but not present in II:3 were selected and these remaining variants were filtered by removing those with a MAF of 0.1% or more in dbSNP142, EVS or ExAC, that are homozygous and those located on the X chromosome. The list is restricted to variants scoring 15 or more when scored with CADD v1.3.

^a^ dbSNP, <http://www.ncbi.nlm.nih.gov/projects/SNP/> ([1](#_ENREF_1));

^b^ Combined Annotation Dependent Depletion (CADD) v1.3, <http://cadd.gs.washington.edu/info> ([2](#_ENREF_2));

^c^ SIFT, http://sift.jcvi.org/ ([3](#_ENREF_3));

^d^ PolyPhen2, http://genetics.bwh.harvard.edu/pph2/ ([4](#_ENREF_4));

| **Type** | **Start (GRCh37)** | **End**  **(GRCh37)** | **Bayes Factor** | | **Reads expected** | | **Reads observed** | | **Reads ratio** | |
| --- | --- | --- | --- | --- | --- | --- | --- | --- | --- | --- |
|  |  |  | **III:3** | **IV:1** | **III:3** | **IV:1** | **III:3** | **IV:1** | **III:3** | **IV:1** |
| Deletion | 4:71388473 | 4:71394475 | 13.6 | 24.4 | 344 | 401 | 185 | 208 | 0.538 | 0.519 |

**Table S3** ExomeDepth output detailing the *AMTN* deletion identified in individuals III:3 and IV:1.


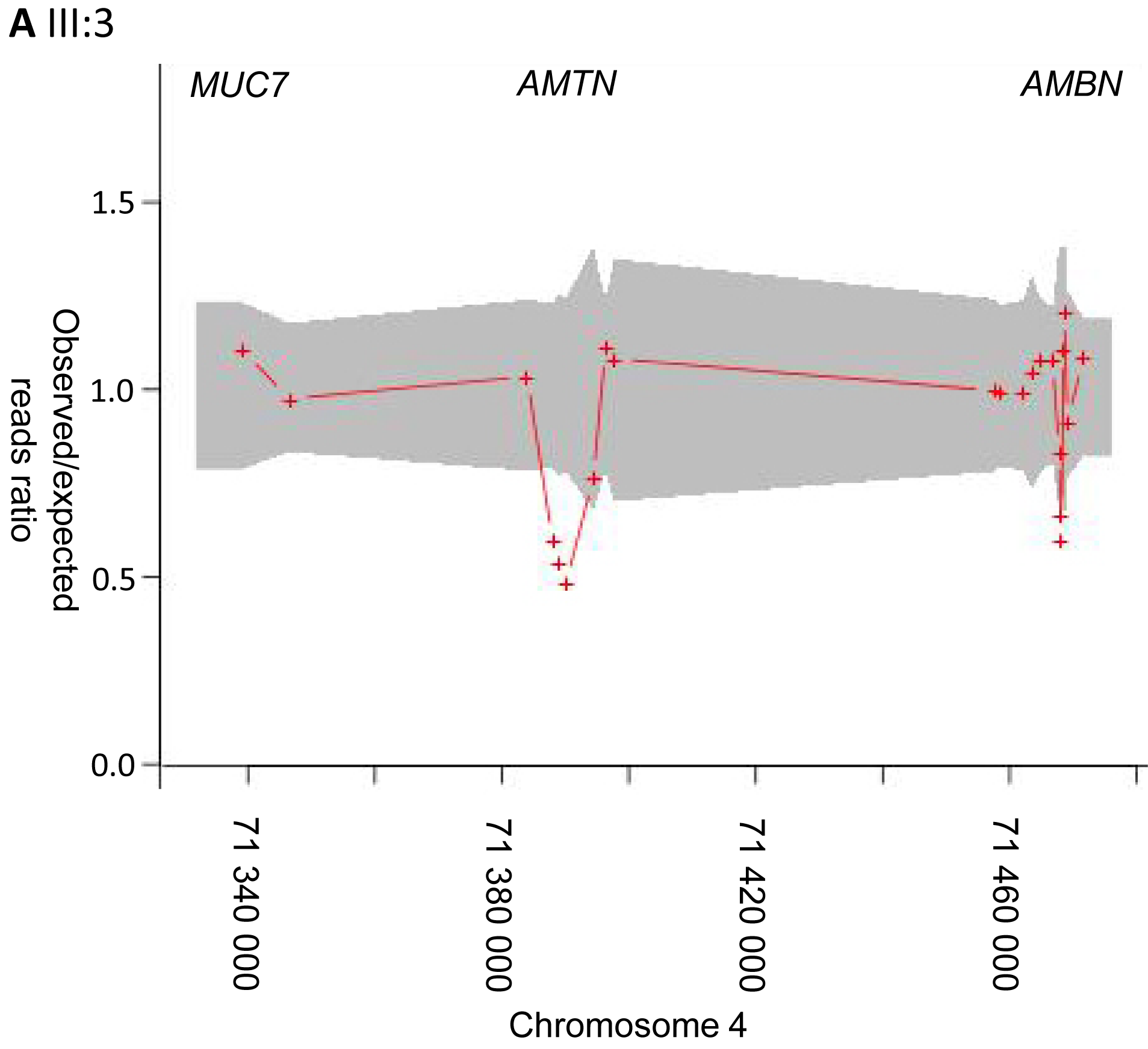


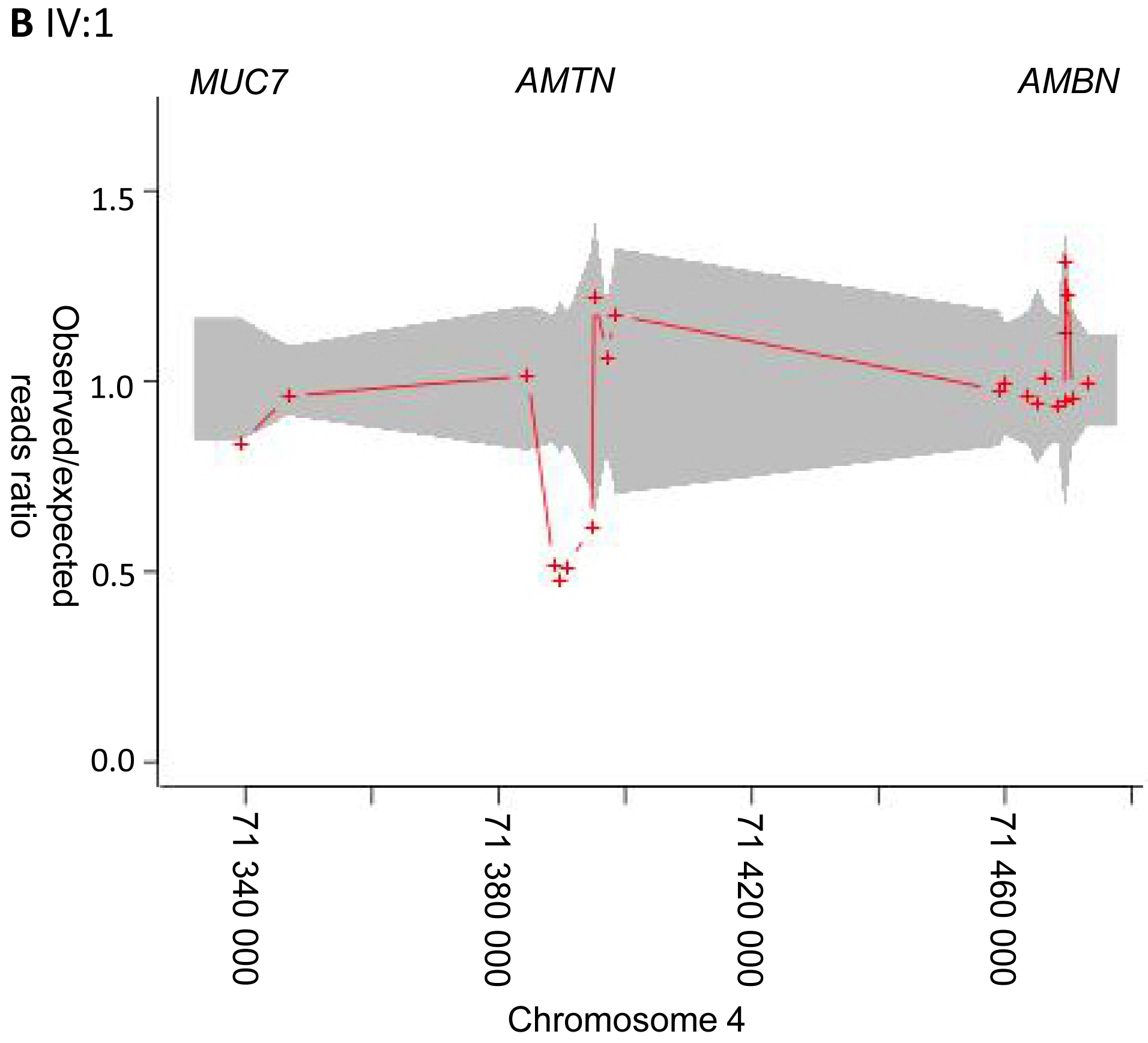


**
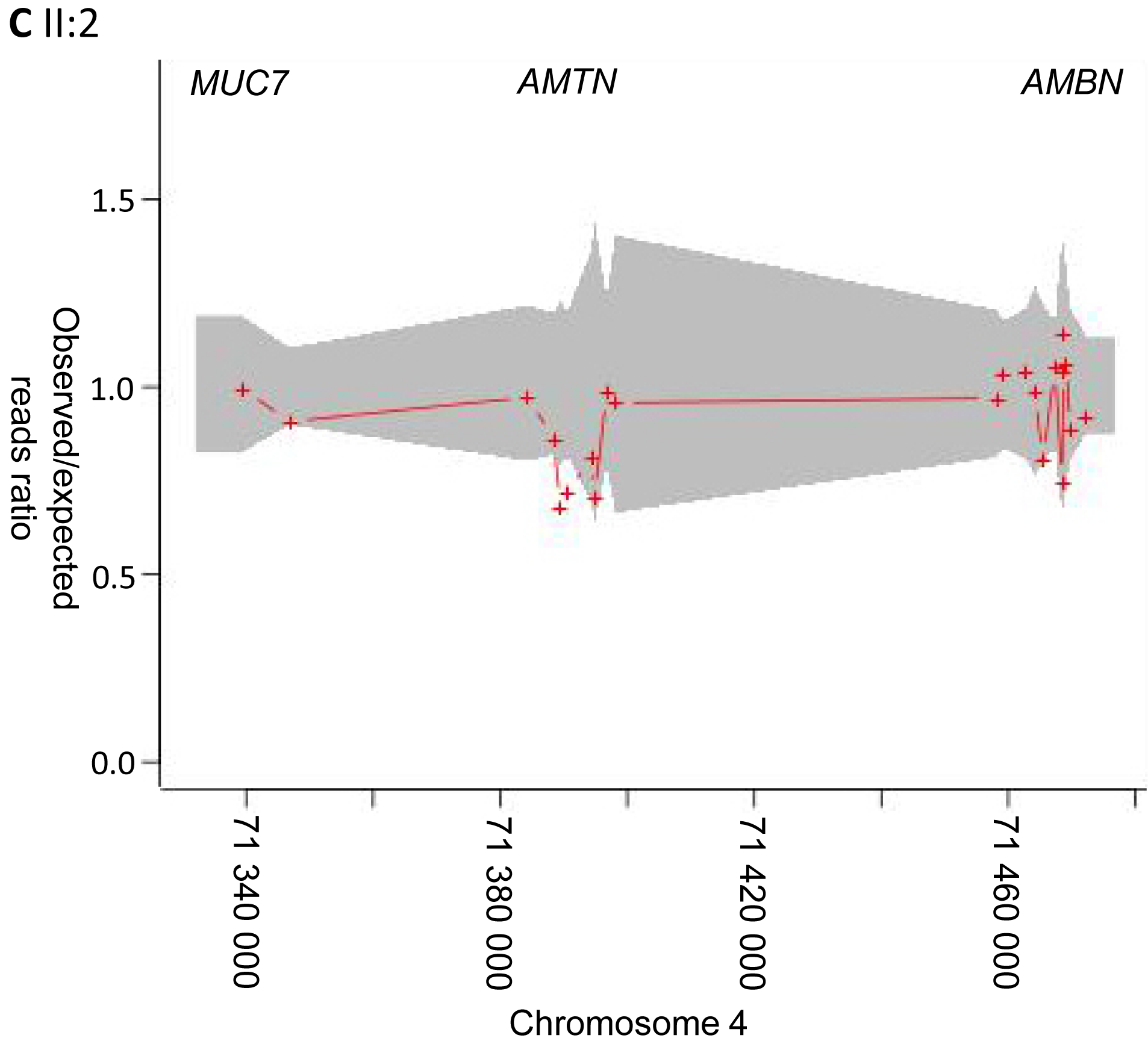
**

**
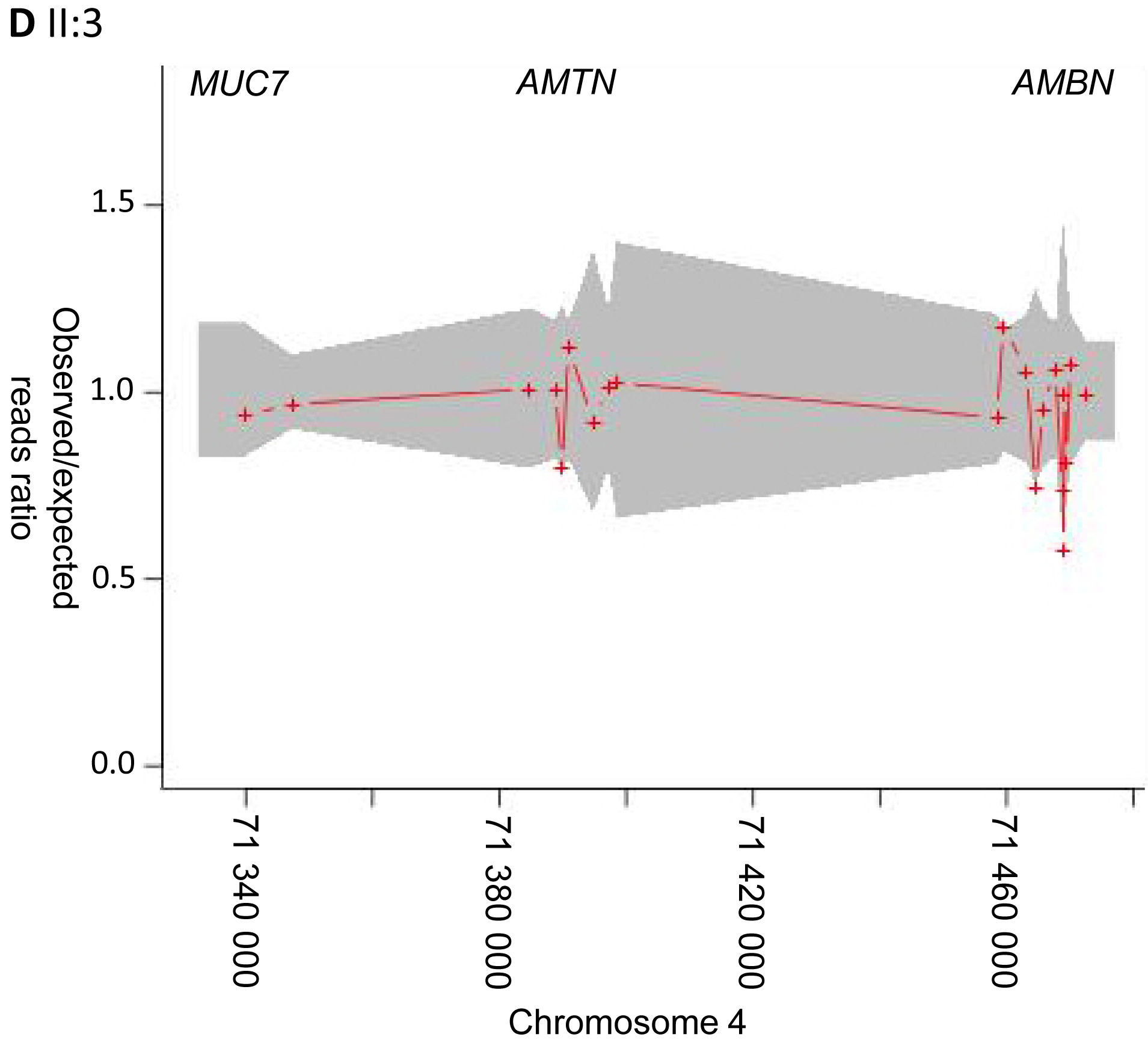
**

**Figure S1** Heterozygous deletion of exons 3, 4, 5 and 6 of *AMTN* gene identified by ExomeDepth in exome sequence data and the corresponding region in family members where the deletion was not identified by the program and / or was not present.

The red crosses indicate the ratio of the observed / expected number of reads for:

A III:3, B: IV:1, C: II:2 and D: II:3. The grey shaded region shows the estimated 99% confidence interval for this ratio in the absence of the CNV call. The presence of four contiguous exons with read count ratios falling outside the confidence intervals is indicative of a heterozygous deletion in sample III:3 (A) and IV:1 (B). For sample II:2 (C), the heterozygous deletion spanning exons 3 to 6 of *AMTN* was not found by ExomeDepth analysis, however, the region lies marginally outside the grey shaded, 99% confidence interval for this sample. The failure to detect the deletion was most likely due to an inadequate number of control sequences from the same sequencing batch since only 5 were available but ExomeDepth requires 10 control sequences for optimal CNV detection ([5](#_ENREF_5)). For sample II:3 (D) the observed calls lie within the confidence limits suggesting the heterozygous deletion is not present in this sample. The number of control sequences used in each case was 11 (A III:3), 9 (B IV:1), 5 (C II:2) and 9 (D II:3).

| Amplicon | Target region  (GRCh37  / hg19) | Forward primer (5′-3′) | Reverse primer (5′-3′) | Size (bp) |
| --- | --- | --- | --- | --- |
| *AMTN* intron 2-intron 6 | chr4:  71385662-71394734 | TGTCCTCCTTGAAAGAAGTTGTC | CAGCATATGATGTGAGACTGCT | 9073  (Wild-Type)  399  (Mutant) |
| *AMTN*  exon 3-exon 6 deletion 5′ breakpoint | chr4:  71385741-71385965 | CAAATGGTTCTTTCACTTCGTCA | CCTGCCCCACTAGCTCTCTA | 225bp  (Wild-Type) |

**Table S4** Primer sequences for Sanger sequencing of *AMTN* deletion and 5′ breakpoint in Wild-Type (primer pair to exons 6-7 used to sequence 3′ breakpoint in Wild-Type, see table S5).

| **Exon** | **Forward primer (5′-3′)** | **Reverse primer (5′-3′)** | **Size (bp)** |
| --- | --- | --- | --- |
| 2 | TTGACCATGTTTCAAGTAGAACTTTT | AGGGCAGGAGAGAGGTAGGT | 246 |
| 3 | AACCTGGATATAAATGGACACAAA | AACGTGTTTTCTTTACCGGTTT | 239 |
| 4 | GAGGATATCCACCACTGACAGA | CACCCATATACATGCTCCCC | 228 |
| 5 | TGTATTTACATAGCAACTCCTTCCTTT | GGGCAAAATTTTCAAGAAGC | 311 |
| 6-7 | CAGTGAGCCAAGACCATGC | TTAGGATTTATGGTCAAAAGAGACTAT | 713 |
| 8 | CTACCCAAACTTGCTCCCCT | CAAGGCAAGACAAAGAACTCCT | 428 |
| 9 | GGATGCTCCTAATCCTGCAA | CCAATGTGTCTCAATCTATTCCA | 211 |

**Table S5** Primer sequences for Sanger sequencing of *AMTN* coding exons in individuals with autosomal dominant AI.

**
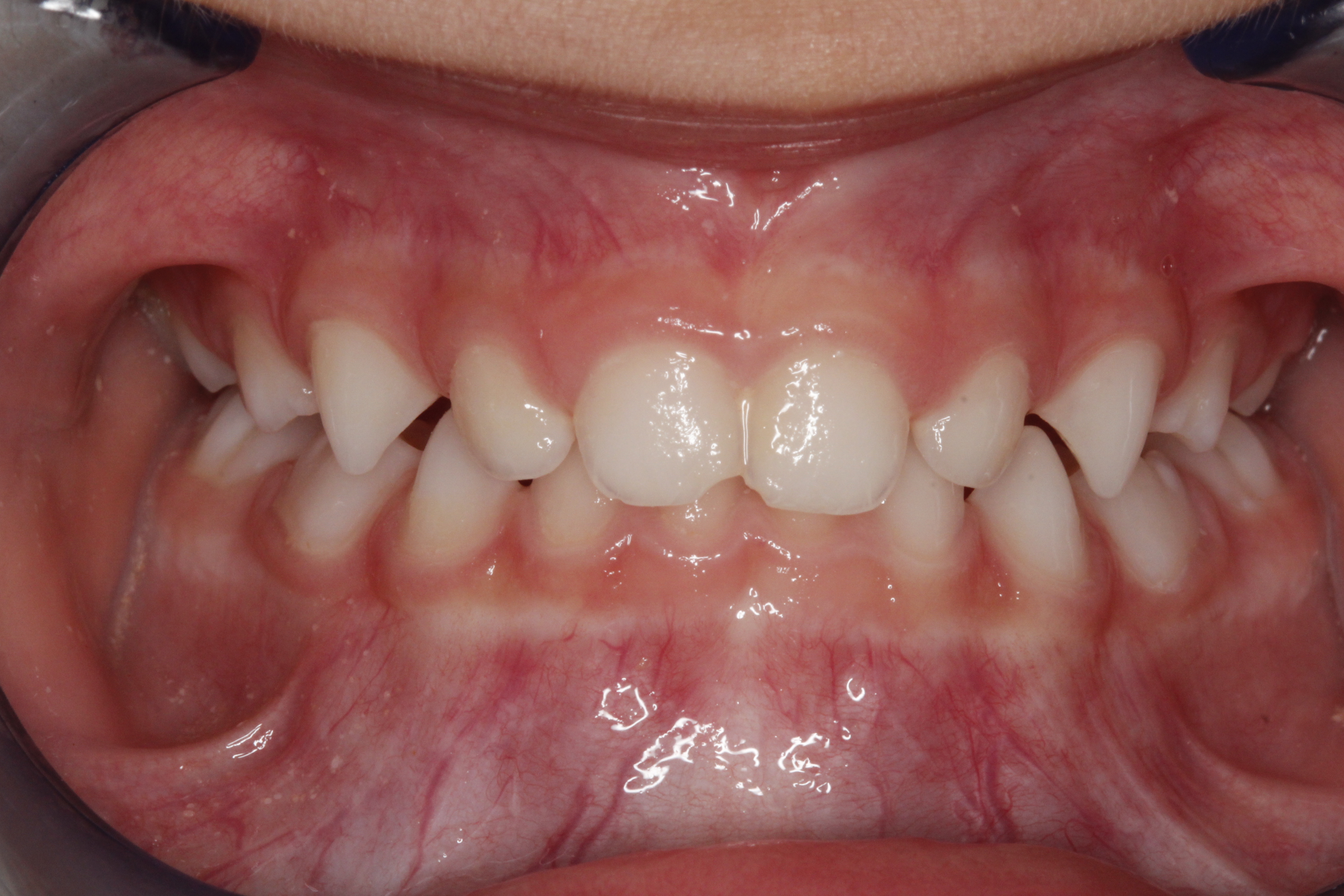
**

**Figure S2** Deciduous dentition of unaffected individual IV:2.

**List of Supplemental Videos**

All videos are in .avi file format.

Video 1: Control tooth 1.

Video 2: Control tooth 2.

Video 3: Affected Individual IV:1 tooth 3.

Video 4: Affected Individual IV:1 tooth 4.

| **Tooth and area analysed** | **Composition (Atomic Mass % ^a^)** | | | | | | **Number of readings** |
| --- | --- | --- | --- | --- | --- | --- | --- |
|  | **Oxygen** | **Calcium** | **Carbon** | **Phosphorus** | **Nitrogen** | **Other^b^**  **(Specified)** |  |
| 4 - 1 | 28.0 ± 2.3 | 11.3 ± 1.0 | 43.6 ± 2.8 | 6.2 ± 0.5 | 11.1 ± 1.9 | 0.2 ± 0.1 (Sodium) | 30 |
| 4 - 2 | 23.8 ± 2.4 | 11.3 ± 1.5 | 46.9 ± 4.9 | 6.1 ± 1.0 | 11.6 ± 2.6 | 0.3 ± 0.2 (Sodium) | 11 |
| 4 - 3 | 47.4 ± 1.1 | 20.3 ± 0.4 | 20.4 ± 1.7 | 11.2 ± 0.6 | 0.0 ± 0.0 | 0.7 ± 0.2 (Sodium) | 10 |
| 4 - 4 | 33.6 ± 1.6 | 4.5 ± 1.9 | 52.1 ± 3.1 | 2.6 ± 1.0 | 7.2 ± 3.6 | N/A | 9 |
| Control 1 | 57.2 ± 0.5 | 19.9 ± 0.4 | 12.4 ± 0.9 | 10.1 ± 0.3 | 0.0 ± 0.0 | 0.5 ± 0.1 (Sodium) | 23 |

**Table S6** EDX elemental analysis of tooth 4 from individual IV:1 and tooth 1 from a control individual.

Values shown are mean values calculated from the number of measurements specified for each area. The error was calculated as 2 x the standard error of the mean. This data has been displayed graphically and in relation to the areas measured in Figure S3.

^a^ Values may not always sum to 100% since values are shown to 1 decimal place.

^b^ For tooth 4, areas 1 and 3, magnesium was also variably present, but the mean composition was less than 0.1% and so it is not included in the table above.

| **Area analysed** | **Composition (Atomic Mass % ^a^)** | | | | | | **Number of readings** |
| --- | --- | --- | --- | --- | --- | --- | --- |
|  | **Oxygen** | **Calcium** | **Carbon** | **Phosphorus** | **Nitrogen** | **Others**  **(Specified)** |  |
| 4-HOCl-1 | 43.3 ± 0.9 | 19.7 ± 1.1 | 21.0 ± 2.3 | 10.9 ± 0.8 | 4.3 ± 0.5 | 0.7 ± 0.1 (Sodium and Magnesium) | 15 |
| 4-HOCl-2 | 39.6 ± 3.1 | 26.1 ± 1.5 | 13.4 ± 5.2 | 14.5 ± 1.1 | 5.5 ± 0.6 | 0.8 ± 0.2 (Sodium and Magnesium) | 10 |

**Table S7** EDX elemental analysis of tooth 4 from individual IV:1 following hypochlorite treatment.

Values shown are mean values calculated from the number of measurements specified for each area. The error was calculated as 2 x the standard error of the mean. This data has been displayed graphically and in relation to the areas measured in Figure S5.

^a^ Values may not always sum to 100% since values are shown to 1 decimal place.


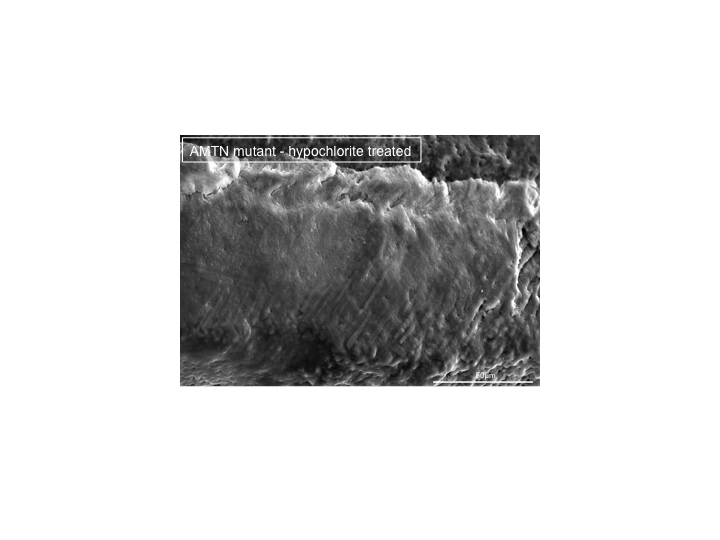


**Figure S3** SEM of enamel of tooth 4 from individual IV:1 following treatment with 6% sodium hypochlorite.

**
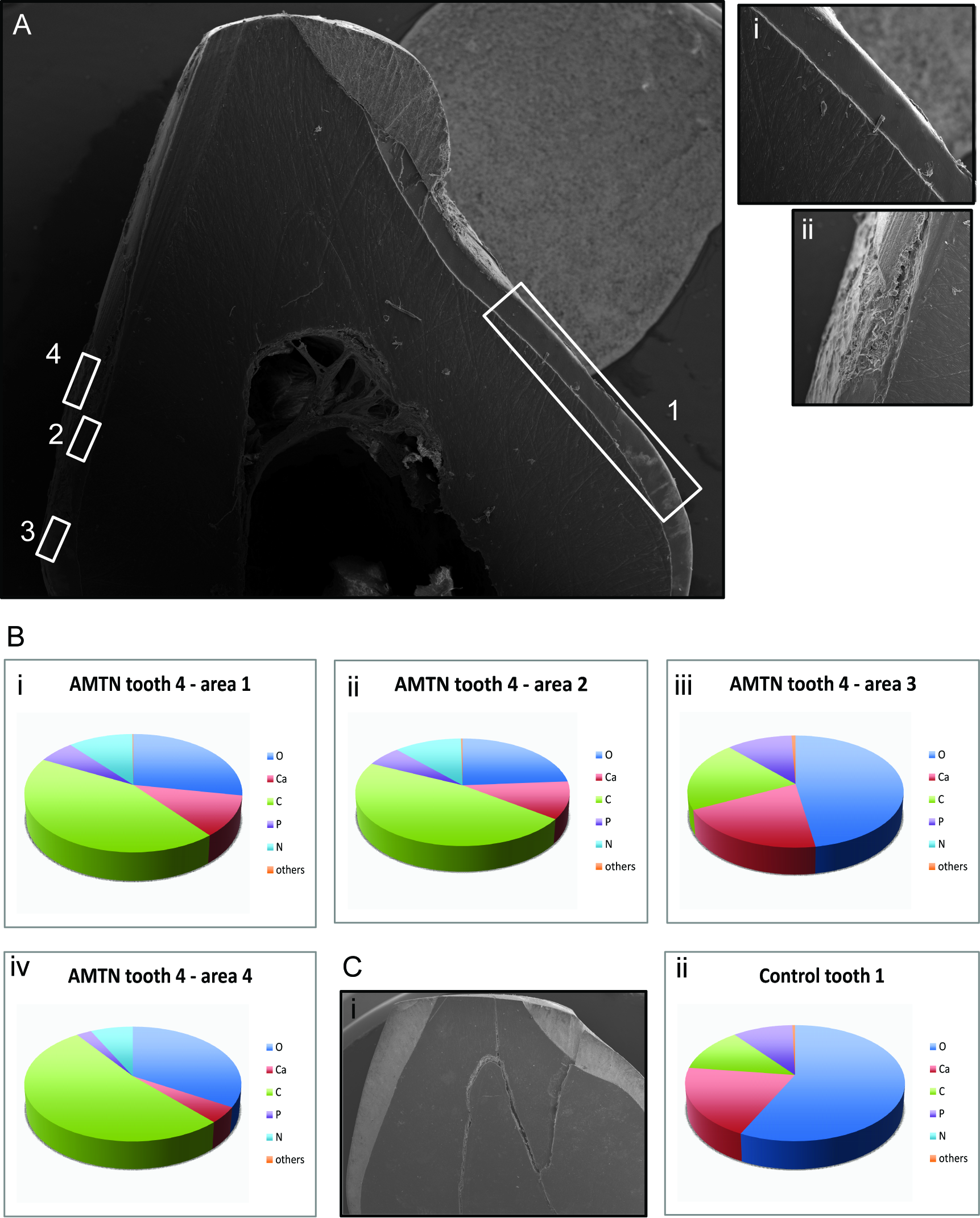
**

**Figure S4** EDX elemental analysis of tooth 4 from individual IV and tooth 1 from a control individual.

A Measurements for tooth 4 were taken from four separate areas of enamel, highlighted by the white boxes. White arrows indicate the areas of enamel that had undergone restoration and therefore where elemental analysis was specifically not undertaken. Panels i and ii detail the appearance of enamel areas 1 and 4 for tooth 4.

B Elemental analysis results are plotted as pie charts, with the percentage atomic contribution of each element to the enamel displayed in separate charts for each area. The number of measurements was as follows: i: Area 1: N=30, ii: Area 2: N=11, iii: Area 3: N=10, iv: Area 4: N=9.

C For comparison, control tooth 1 was also analysed at points throughout the area of enamel shown in panel i. Panel ii shows elemental analysis results for control tooth 1 plotted as before, N=23.

In all cases, the “others” section for each elemental analysis pie chart refers to the elements Na and/or Mg that were found to contribute <1% collectively to the enamel.

For a detailed breakdown of the percentage contributions of each element to each of the tooth regions analysed, see Table S6.


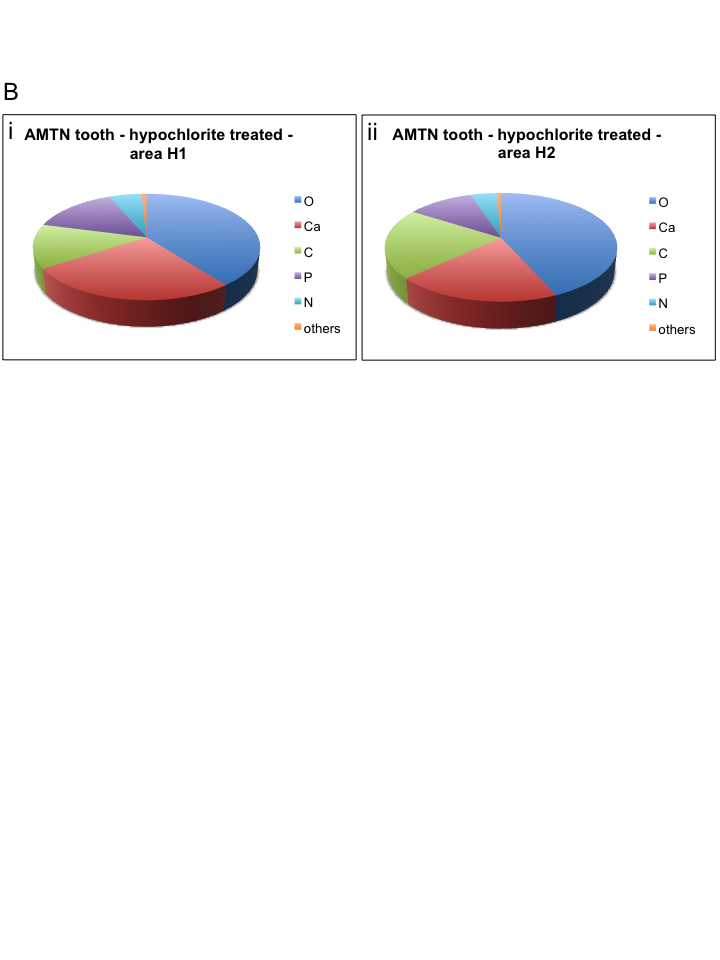

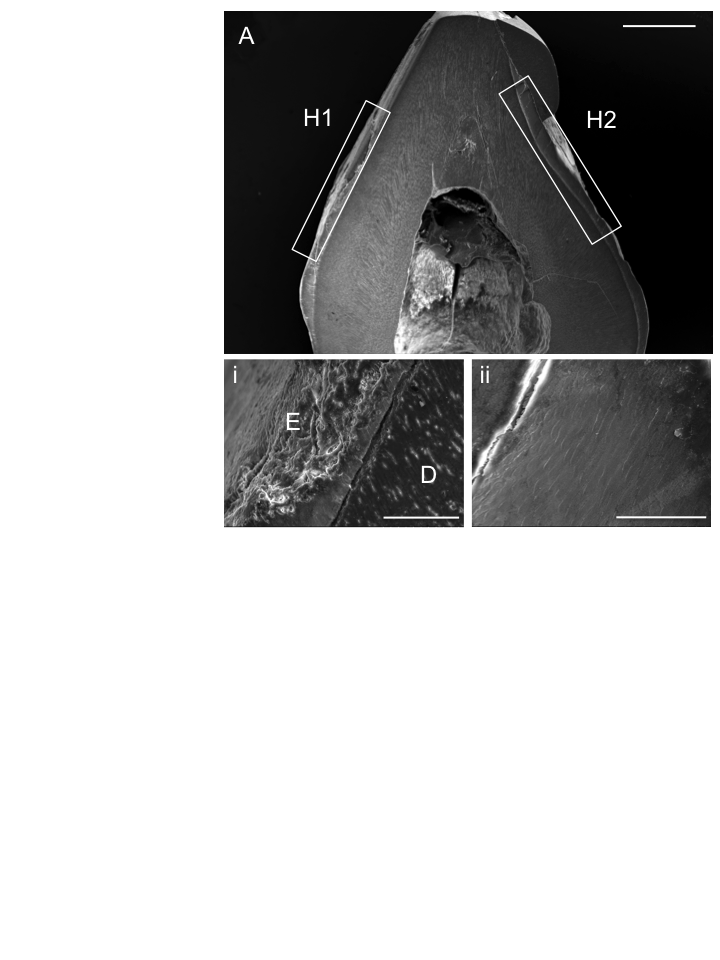


**Figure S5** EDX elemental analysis of tooth 4 from individual IV following hypochlorite treatment.

A Measurements for tooth 4 following hypochlorite treatment were taken from two separate areas of enamel, highlighted by the white boxes. Elemental analysis of areas of enamel that had undergone restoration (previously highlighted in Figure S4) was deliberately avoided. Panels i and ii detail the appearance of the enamel (E and dentine (D), if ) within areas H1 and H2. The image for area H2 shows enamel only.

B Elemental analysis results are plotted as pie charts, with the percentage atomic contribution of each element to the enamel displayed in separate charts for each area. The number of measurements was as follows: i: Area H1: N=15, ii: Area H2: N=10.

In all cases, the “others” section for each elemental analysis pie chart refers to the elements Na and Mg that were found to contribute <1% collectively to the enamel.

For a detailed breakdown of the percentage contributions of each element to each of the tooth regions analysed, see Table S7.

Scale bars represent 1mm (A), 100μm (B) and 50μm (C).

**References**

1. Sherry, S.T., Ward, M.H., Kholodov, M., Baker, J., Phan, L., Smigielski, E.M. and Sirotkin, K. (2001) dbSNP: the NCBI database of genetic variation. *Nucleic Acids Res.*, **29**, 308-311.

2. Kircher, M., Witten, D.M., Jain, P., O'Roak, B.J., Cooper, G.M. and Shendure, J. (2014) A general framework for estimating the relative pathogenicity of human genetic variants. *Nat. Genet.*, **46**, 310-315.

3. Kumar, P., Henikoff, S. and Ng, P.C. (2009) Predicting the effects of coding non-synonymous variants on protein function using the SIFT algorithm. *Nat. Protoc.*, **4**, 1073-1081.

4. Adzhubei, I.A., Schmidt, S., Peshkin, L., Ramensky, V.E., Gerasimova, A., Bork, P., Kondrashov, A.S. and Sunyaev, S.R. (2010) A method and server for predicting damaging missense mutations. *Nat. Methods*, **7**, 248-249.

5. Plagnol, V., Curtis, J., Epstein, M., Mok, K.Y., Stebbings, E., Grigoriadou, S., Wood, N.W., Hambleton, S., Burns, S.O., Thrasher, A.J. *et al.* (2012) A robust model for read count data in exome sequencing experiments and implications for copy number variant calling. *Bioinformatics*, **28**, 2747-2754.
